# Ulinastatin attenuates capillary leakage and suppresses FoxO1-dependent angiopoietin-2 in sepsis-associated acute lung injury via PI3K pathway

**DOI:** 10.1371/journal.pone.0348261

**Published:** 2026-04-28

**Authors:** Yating Lv, Yanfei Meng, Haiying Rui, Tongqin Li, Jing Zhang, Jie Liu, Xin Ma, Jiaqi Wang, Yamin Yuan, Yujing Jiang, Xiaoxi Yan, Miaobo Li, Xiaorong Dong, Bei Zhang, Li Ma

**Affiliations:** Department of Critical Care Medicine of the Second Hospital & Clinical Medical School, Lanzhou University, Lanzhou City, Gansu Province, People’s Republic of China; Stanford University, UNITED STATES OF AMERICA

## Abstract

Sepsis-associated acute lung injury (SALI) is characterized by endothelial barrier dysfunction and capillary leakage. Ulinastatin (UTI), a serine protease inhibitor with recognized clinical benefits in sepsis, has been reported to protect endothelial function, but the underlying mechanisms remain incompletely defined. This study investigated the protective effects of UTI against SALI and its specific mechanism of action. We found that UTI attenuated lung injury and endothelial dysfunction in both cecal ligation and puncture (CLP)-induced septic rats and LPS-stimulated human umbilical vein endothelial cells (HUVECs). UTI treatment reduced the expression of angiopoietin-2 (Ang-2), a key mediator of vascular destabilization, and exerted similar protective effects on endothelial function as dexamethasone (DEX). Mechanistically, UTI was demonstrated to have a stable interaction and favorable binding affinity to PI3K by docking and activating the PI3K/Akt signaling pathway. This led to phosphorylation and subsequent nuclear export of the transcription factor FoxO1, thereby suppressing FoxO1-dependent Ang-2 transcription. The protective effects of UTI on capillary leakage and junctional protein integrity were abolished by the PI3K inhibitor wortmannin. In conclusion, our findings demonstrate that UTI alleviates SALI by disrupting an Ang-2-mediated vicious cycle via the PI3K/Akt/FoxO1 pathway, revealing a novel mechanistic insight into its therapeutic action against sepsis-induced vascular leakage.

## Introduction

Sepsis ranks among the leading causes of mortality globally [1]. According to statistics from 2020, this condition affected 48.9 million people worldwide and contributed to 11 million deaths, accounting for approximately 20% of all fatalities globally [2]. The hallmark features of sepsis are microcirculatory dysfunction and endothelial hyperpermeability, which subsequently lead to the leakage of protein-rich fluid into the interstitial space. Clinical phenotypes of excessively fluid extravasation are hypovolemia, progressive extravascular edema, and hypoperfusion, known as capillary leak syndrome, which can be driven by sepsis [3]. Nevertheless, there is no specific pharmacological therapy currently used to attenuate capillary leakage [4]. Sepsis-associated acute lung injury (SALI) is featured as widespread pulmonary endothelial barrier dysfunction [5], which manifests by endothelial glycocalyx shedding, dysregulation of the cytoskeleton, and damage to intercellular junctions such as zonula occludens-1 (ZO-1), occludin, and vascular endothelial-cadherin (VE-cadherin) [6]. The endothelial barrier dysfunction facilitates circulating fluids and immune cells into alveoli, thereby significantly exacerbating the SALI [7]. Although preclinical studies have been conducted for decades, translating these findings into effective treatments or targeted drug therapies remains challenging [8].

Angiopoietin-2 (Ang-2) has been identified as a critical mediator of vascular pathology during sepsis [9]. In the context of sepsis, a massive release of Ang-2 stored within endothelial Weibel-Palade bodies occurs, leading to direct induction of cytoskeletal rearrangement, disruption of intercellular junctions, and endothelial glycocalyx shedding [10,11]. Ang-2 competitively binds to the Tie-2 receptor on endothelial cells with Angiopoietin-1 (Ang-1), exerting an antagonistic effect. During vascular quiescence, Ang-1 agonists Tie-2 receptor, supporting vascular stability and activating the downstream signaling pathway phosphatidylinositol 3-kinase (PI3K) /protein kinase B (PKB/AKT) [12], leading to phosphorylation and nuclear export of transcription factor Forkhead box protein O1 (FoxO1), ultimately diminishing Ang-2 gene expression [13]. When endothelial cells are exposed to inflammation, the release of pre-formed Ang-2 antagonizes Tie-2 to inhibit the downstream pathway PI3K/Akt, enabling FoxO1 to remain in the nucleus. Reduced nuclear export of FoxO1 leads to elevated Ang-2 levels, setting off a vicious positive feedback loop of vascular leakage [11]. Breaking this vicious cycle of endothelial Ang-2 production becomes an essential therapeutic target in sepsis-associated capillary leakage.

Ulinastatin (UTI) is a significant protease inhibitor extracted from fresh human urine, and it is commonly used in the treatment of pancreatitis. In recent years, it has been widely applied in septic patients to decrease the 28-day mortality [1]. UTI-combination therapy in Acute Respiratory Distress Syndrome (ARDS) patients significantly improves the lung function [14]. Previous research indicated that UTI exerted the protective effects by inhibiting systemic inflammation and anti-oxidative stress in septic models [15]. Strong evidence demonstrated that UTI attenuated pulmonary capillary hyperpermeability by protecting endothelial tight junctions, resulting in improved SALI [16–18]. Notably, UTI attenuated LPS-induced acute lung injury by inhibiting the expression of Ang-2 in juvenile rats [19]. Recently, it has been reported that UTI served as a multifactorial Ang-2 inhibitor through blocking the nuclear entry of FoxO1/p-STAT3, disrupting exocytosis of Ang-2, and directly binding with Ang-2, also restoring the phosphorylation of the Tie-2/Akt pathway, eventually ameliorating the endothelial barrier dysfunction and treating septic cardiomyopathy [20]. Nevertheless, the precise mechanism by which UTI suppresses Ang-2 in sepsis-associated acute lung injury remains unclear. In particular, whether UTI alleviates vascular leakage by modulating the PI3K/Akt/FoxO1/Ang-2 signaling axis has not been fully elucidated. Further investigation is required to determine whether UTI exerts protective effects through breaking the vicious cycle of Ang-2 expression.

Here, we established septic models to systematically investigate the therapeutic efficacy of UTI in treating capillary leakage during SALI. We suggested that the underlying molecular mechanism of how UTI broke the vicious cycle of Ang-2 production.

## Materials and methods

### Animals

Male Wistar rats, weighing 200 ± 20 g, were purchased from the Animal Experimental Research Center of Lanzhou University. We followed the guidelines of the ARRIVE guidelines (Reporting of In Vivo Experiments). The protocol for experimental animal was approved by the Experimental Animal Welfare Ethics Committee of Lanzhou University Second Hospital (ethics number: D2024-773). The rats (mean weight 200g) were euthanized via inhaled sevoflurane (5% induction), followed by bilateral pneumothorax to ensure death. Researchers involved in animal handling and surgical procedures received specialized training in animal care and laboratory techniques provided by the Gansu Provincial Technical Center for Laboratory Animal Industry. The rats were housed under standard conditions with a 12-h light/dark cycle and had free access to food and water.

### CLP‐induced polymicrobial sepsis model and treatment

Firstly, animals were randomly allocated to each group according to a random number table, and group allocation was performed by an independent investigator, and coded labels were used to conceal group assignments during outcome assessment and data analysis. The sample size for each group was determined based on previous studies in our group, with consideration of ethical principles to minimize animal use.

After a 12-h fasting period with free access to water, a polymicrobial sepsis model was induced by the cecal ligation and puncture (CLP), which is widely recognized as a clinically relevant model because it reproduces major features of human sepsis, including systemic inflammation, endothelial injury, capillary leakage, and organ dysfunction. Anesthesia was induced by intraperitoneal injection of 2% sodium pentobarbital at 20 mg/kg body weight. The ventral abdomen was shaved, disinfected, and opened through a midline incision. The cecum was exteriorized, and approximately the distal half was ligated in proximity to its free end. A single perforation was then made through the ligated segment with a 21-gauge needle, and a small amount of fecal material was gently extruded to ensure patency. Based on the severity of ligation and puncture, this protocol was considered to induce a moderate-to-severe sepsis model. Subsequently, the cecum was returned to its original position within the abdominal cavity, and the musculature and skin were sutured in separate layers. Postoperatively, warm saline was administered subcutaneously, and the animals were returned to their cages with free access to standard chow and water. Sham-operated rats underwent laparotomy and cecal manipulation, but without cecal ligation or puncture.

After surgery, 5 ml of saline was intraperitoneally injected to restore the hydration of the operated rats. The rats were injected intraperitoneally with UTI (100 KU/kg, Techpool Bio-Pharma, China) was administered at 0 h, 6 h, and 12 h after CLP, this dosing strategy was selected with reference to previous rat CLP studies using 100 kU/kg UTI and repeated early post-CLP administration schedules, and was adapted to maintain pharmacological intervention during the early inflammatory phase of sepsis [21]; the PI3K inhibitor wortmannin (0.7 mg/kg, Solarbio, China) was administered 1h before and 6h after CLP; dexamethasone (DEX, 0.5 mg/kg, Aladdin, China) was administered at 0h after CLP.

For pathophysiological comparison, rats underwent CLP surgery and were monitored every 4 hours for clinical signs of distress. Humane endpoints were established to minimize suffering; animals exhibiting severe signs of sepsis (labored breathing, complete loss of mobility, or lack of response to external stimuli) were to be euthanized immediately. In this study,48 rats were used; 30 rats survived to the planned 24-hour endpoint, and 18 rats required early euthanasia. At 24h after CLP surgery, blood and histological samples were collected under deep anesthesia via inhaled sevoflurane. Blood drawn via the right ventricle was used for cytokine detection and various laboratory tests. The lung tissues were snap frozen or fixed with formalin for further examination and then stored at −80 °C. Investigators were blinded to the group allocation for the analysis.

### Cell culture and treatment

Human umbilical vein vascular endothelial cells (HUVECs) were purchased from ScienCell and were cultivated in Dulbecco’s modified Eagle medium (DMEM) containing 1% endothelial cell-derived growth factor (ECGS, ScienCell, USA), 10% fetal bovine serum (SinoBiological, China), and 1% streptomycin and penicillin (Biosharp, China). Cells were placed in an incubator at 37°C, 5% CO2, and 95% humidity. To mimic the inflammatory environment in vitro, the HUVECs were stimulated with 10 μg/mL LPS (L8880, Escherichia coli 055: B5, Solarbio, China) for 24h. In separate experiments, HUVECs were pre-treated with UTI (10 KU/mL, Techpool Bio-Pharma, China) for 1h, a PI3K inhibitor wortmannin (1 μM, W8030, Solarbio, China) or dexamethasone (10 μM, Solarbio, China) for 1h, followed by LPS stimulation for 24h.

### Cell viability assay

Cell viability was assessed using a Cell Counting Kit-8 (CCK-8) assay kit (M4839, Abmole, China). HUVECs were cultured in 96-well plates and exposed to varying concentrations of UTI (1,5,10,20,40 KU/mL). Subsequently, CCK-8 reagent was added to each well and incubated at 37°C for 2 h in the absence of light.

### Evaluation of endothelium barrier permeability

HUVECs were cultured using the Transwell system (0.4 μm pore size polyester membrane inserts, 14312, Labselect, China). Measurement of FITC-FD40 (F491425, Aladdin, China) across the endothelium was used to evaluate endothelial barrier permeability. The HUVECs were treated to remain unchanged from the previous instance. We added 0.1 mg/mL FITC-FD40 to the upper inserts, and an equal amount of serum-free medium was added to the lower compartments of the Transwell system for 60 minutes. Fluorescence across the upper inserts was measured respectively at excitation and emission wavelengths of 492 and 518 nm.

### Immunofluorescence staining

HUVECs were washed with PBS, fixed in 4% PFA, and permeabilized with 0.5% Triton X-100 (T8200, Solarbio, China) or not. Then blocked with 5% bovine serum albumin (BS114, Biosharp, China) for 1h at room temperature. Cells were then incubated with primary antibodies diluted in primary antibody against ZO-1 (21773–1-AP, Proteintech, Wuhan, China) and FoxO1 (18592–1-AP, Proteintech, China) overnight at 4°C. Cells were slowly rewarmed for 1 h at room temperature on the following day and washed with PBS, then incubated for 1 h with fluorescent secondary (1:500, P0179, Beyotime Biotechnology, China) antibody. Slides were mounted using antifade mounting medium containing DAPI (BL739, Biosharp, China) for nuclear staining with a microscope cover glass (803400130, CITOGLAS, China). Images were acquired using a Upright fluorescence microscope (BX53 + DP74, Olympus, Japan) and analysed by ImageJ.

### Enzyme-linked immunosorbent assay

Following the instructions of manufacturers, the TNF-α, IL-6, and angiopoietin-2 levels in rat serum were measured using a TNF-α ELISA kit (RX2D310636, RUIXIN BIOTECH, China), the IL-6 ELISA kit (RX2D302196, RUIXIN BIOTECH, China), and the angiopoietin-2 (JRX302196R, RUIXIN BIOTECH, China). The TNF-α and IL-6 levels in cell supernatants were measured using by TNF-α ELISA kit (KE00367, Proteintech, China) and the IL-6 ELISA kit (KE00385, Proteintech, China).

### Western blot analysis

The methodology for Western blot analysis involved denaturing HUVECs and lung tissues. Lung tissues and HUVECs were lysed using RIPA buffer (R0010, Solarbio, China) containing protease and phosphatase inhibitors (P0100, Solarbio, China). Protein concentration was quantified using the BCA protein assay kit (BL521A, Beyotime Biotechnology, China). An equal amount of protein was added to a sodium dodecyl sulfate-polyacrylamide gel and separated by electrophoresis. Proteins were transferred onto PVDF membranes (Millipore). The membranes were then blocked in 0.1% Tween-20 containing 5% skim milk or 5% BSA. Subsequently, they were incubated with the respective primary antibodies at 4 °C overnight, followed by incubation with horseradish peroxidase-conjugated secondary antibodies (GB23303, Servicebio, China) for 1 h at room temperature. Chemiluminescent signals were developed using an ECL detection system (BL520A, Biosharp, China), and band densities were analysed with ImageJ software (NIH). The following primary antibodies were used: ZO-1 (1:10000, 21773–1-AP, Proteintech, China), VE-cadherin (1:2000, BA3032, Boster, China), occludin (1:10000, 13409–1-AP, Proteintech, China), Akt (1:1000; 8242S, Cell Signaling Technology, USA), p-Akt (1:1000, 4060, Cell Signaling Technology, USA), FoxO1 (1:1000, 18592–1-AP, Proteintech, China), p-FoxO1 (1:1000, 84192, Cell Signaling Technology, USA), Angiopoietin-2 (1:5000, ET1705−6, HuaBio, China), β-TUBULIN (1:10000, ET1602−4, HuaBio, China), β-actin (1:10000, EM21002, HuaBio, China).

### Histological and immunohistochemical staining

Paraffin-embedded rat lung tissue sections were stained with H&E for blinded histopathologic assessment as described previously [22]. For Immunohistochemical staining, the primary antibody was ZO-1 (Proteintech, China), occludin (Proteintech, China), VE-cadherin (Boster, China) and angiopoietin-2 (HuaBio, China). The sections were then treated with a secondary HRP-linked antibody (Servicebio, China) at 4°C overnight, followed by incubation with biotinylated secondary antibody for 30 min at 37°C, and finally visualized with a 3,3’-diaminobenzidine solution and counterstained with hematoxylin. The images were taken using an optical microscope (TissueFAXS Plus, Austria).

### Assessment of Evans blue extravasation

Evans blue dye (EB) extravasation assay was used to evaluate the rat pulmonary vascular permeability. EB (20 mg/kg, E8010, Solarbio, China) in 1mL of 0.9% saline was injected into the rat abdominal cavity 2h before sacrifice. The removal of intravascular Evans blue (EB) was achieved through the transcardial perfusion of rats with 0.9% saline prior to the collection of lungs. The lungs were then weighed, homogenized, and incubated in formamide (1 mL per 100 mg of tissue) at 37°C for 24 hours. Following this incubation period, the resulting supernatants were collected and subsequently measured using a spectrophotometer at a wavelength of 620 nm. The EB content was calculated from a standard curve and expressed as µg of Evans blue per 100 mg of lung tissue.

### Lung wet-to-dry weight ratio

Immediately after resection, the lung tissue sample was weighed to obtain its wet weight. Subsequently, the sample was placed in a 60°C oven for drying for 48 hours. The tissue was weighed again to determine its dry weight. Finally, the wet-to-dry ratio was calculated by dividing the wet weight by the dry weight.

### Molecular docking

The 3D structure of ulinastatin was prepared and coordinates determined using the Ligprep module of Schrödinger software based on the OPLS_4 force field. The Epik module was employed to identify all possible stereoisomers and associated protonation states. The protein structure of PI3K was obtained from the Protein Data Bank (PDB, ID: 8TWY), utilizing the highest resolution 9CMK crystal structure. Protein preparation was performed using the Protein Preparation Wizard module of Schrödinger software. Perform operations including bond order assignment, hydrogenation, and missing side-chain completion. Subsequently, remove water molecules and cofactors from the protein. Optimise the hydrogen bond network, then minimise the protein energy using the OPLS_4 force field. Perform molecular docking using the Glide module within Schrödinger.

### Statistical analysis

The data are expressed as the mean ± standard deviation (SD) from a minimum of three independent experiments and were analyzed using GraphPad Prism 9.5. The normality of continuous variables was assessed using the Shapiro–Wilk test and an examination of Q–Q plots. The homogeneity of variance was evaluated using the F-test or the Brown–Forsythe/Bartlett tests. For normally distributed data with similar variances, between-group differences were analyzed using unpaired two-tailed Student#39;s t-tests (two groups) or one-way ANOVA with Tukey#39;s post hoc test (three or more groups). In instances where variances were found to be unequal, the Welch#39;s t-test or the Welch/Brown–Forsythe ANOVA was implemented, subsequently followed by the Dunnett#39;s T3 test. Non-normally distributed data were analyzed with the Mann–Whitney U test (two groups) or the Kruskal–Wallis test with Dunn#39;s multiple comparisons (three or more groups). A P value < 0.05 was considered statistically significant. For all statistical analyses, n represents biological replicates, including independent rats in animal experiments and independent samples in cell experiments. Technical replicates were not treated as independent observations and were not included in the statistical analysis.

## Results

### UTI attenuates sepsis-associated acute lung injury (SALI) and endothelial barrier dysfunction in vivo and vitro

**Fig 1:**
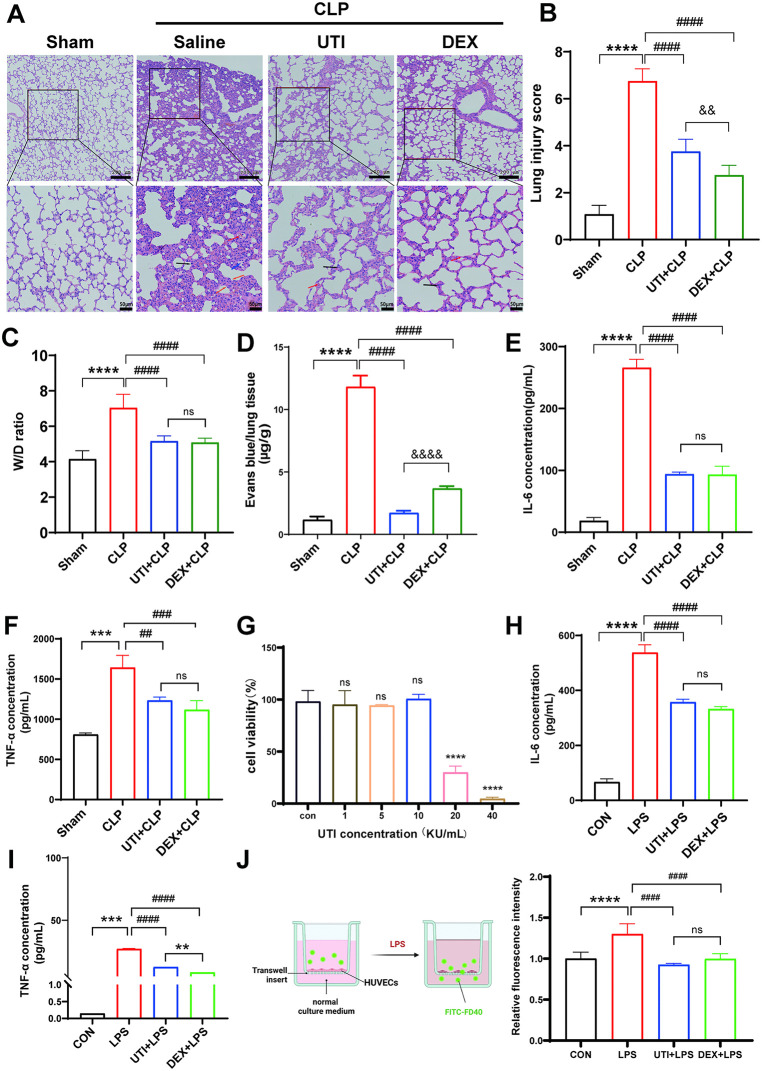
Ulinastatin (UTI) attenuated sepsis-associated acute lung injury (SALI) and endothelial barrier dysfunction in vivo and vitro. **(A-B)** Sepsis is induced by cecal ligation and puncture (CLP), treated with UTI (100 KU/kg, i.p.) or dexamethasone (DEX, 0.5 mg/kg, i.p.). The rats were harvested 24h after surgery. Representative images of H & E staining of rat lung tissue section (upper scale bar = 200μm, bottom panel = 50μm). H & E stainings indicate signs of edema (blue arrows), hemorrhage (red arrows), inflammation (black arrows), and alveolar septal thickening (yellow arrows) from the cross-section of the lung (n = 6). Quantification of lung injury scores was determined in panel **B. (C-D)** Lung wet-to-dry weight ratio and Evans Blue dye extravasation were determined in lung tissues (n = 6). **(E-F)** The levels of TNF-α and IL-6 in serum were measured by ELISA (n = 6). **(G)** HUVECs were exposed to UTI for 24h, and cell viability was determined by CCK8 (n = 6). **(H-I)** HUVECs were pretreated with UTI (10 KU/mL) or DEX (10 μM) for 1 hour before LPS stimuli for 24 h. Levels of IL-6 and TNF-α in the supernatants of HUVECs were examined by ELISA (n = 6). **(J)** Pattern diagram for the Transwell model. The endothelial permeability was estimated by the fluorescence signal intensity of FITC-FD40 from the Transwell system (n = 6). All data were expressed as the mean  ±  SD. ns = no significance, ^*^p  <  0.05, ^**^p  <  0.01, ^***^p  <  0.001, and ^****^p  <  0.0001. ^*##*^*p*  <  0.01, ^*###*^*p*  <  0.001, and ^*####*^*p* ＜ 0.0001.

Ulinastatin (UTI), a urinary trypsin inhibitor derived from human urine, is widely used to treat acute inflammatory diseases. Firstly, we established a sepsis model by performing cecal ligation and puncture (CLP) to examine the protective effect of UTI on SALI. H&E staining showed that CLP caused such a substantial increase in vascular leakage characterized by edema, hemorrhage, alveolar septal thickening, and inflammatory cell infiltration, while the increase was abrogated in the UTI or dexamethasone (DEX) group (Fig 1A, 1B). To determine pulmonary vascular permeability, we measured the wet/dry (W/D) ratio and the relative Evans Blue leakage content. CLP significantly increased the lung W/D ratio and aggravated Evans Blue leakage. UTI or DEX obviously reduced lung W/D ratio, which was accompanied by a reduction in Evans Blue leakage (Fig 1C, 1D). Furthermore, compared with the control group, the inflammatory cytokines (TNF-α and IL-6) increased significantly in the septic serum. These markers were reduced by UTI treatment, with the therapeutic effect being approximately equivalent to that of the DEX group (Fig 1E, 1F)**.**

We next determined the impact of UTI on endothelial barrier function in vitro. The impact of UTI on the survival and growth of HUVECs was assessed by the CCK-8 assay. The results showed that UTI exhibited minimal toxicity towards HUVECs at concentrations of 10 KU/mL (Fig 1G). We pretreated HUVECs with UTI for 1h, followed by exposure to LPS (10 μg/mL) for 24h. As shown in Fig 1H-I, LPS led to a significant upregulation of pro-inflammatory cytokines IL-6 and TNF-α expression levels in HUVECs. In contrast, UTI and DEX treatment reduced the expression of these inflammatory factors. Endothelial barrier dysfunction is a key pathological feature of capillary leakage. To test the effect of UTI on the endothelial barrier function, we employed the Transwell system, which was achieved by detecting the fluorescence signal intensity of FITC-FD40 in the lower chamber. We found that UTI attenuated LPS-induced augmentation in endothelial permeability significantly (Fig 1J)**.** Additionally, it indicated that DEX exerted similar protective effects on endothelial permeability as UTI. The above results showed that UTI suppressed the SALI and endothelial barrier dysfunction in vivo and in vitro.

### UTI improved endothelial barrier function by protecting the junction proteins

**Fig 2:**
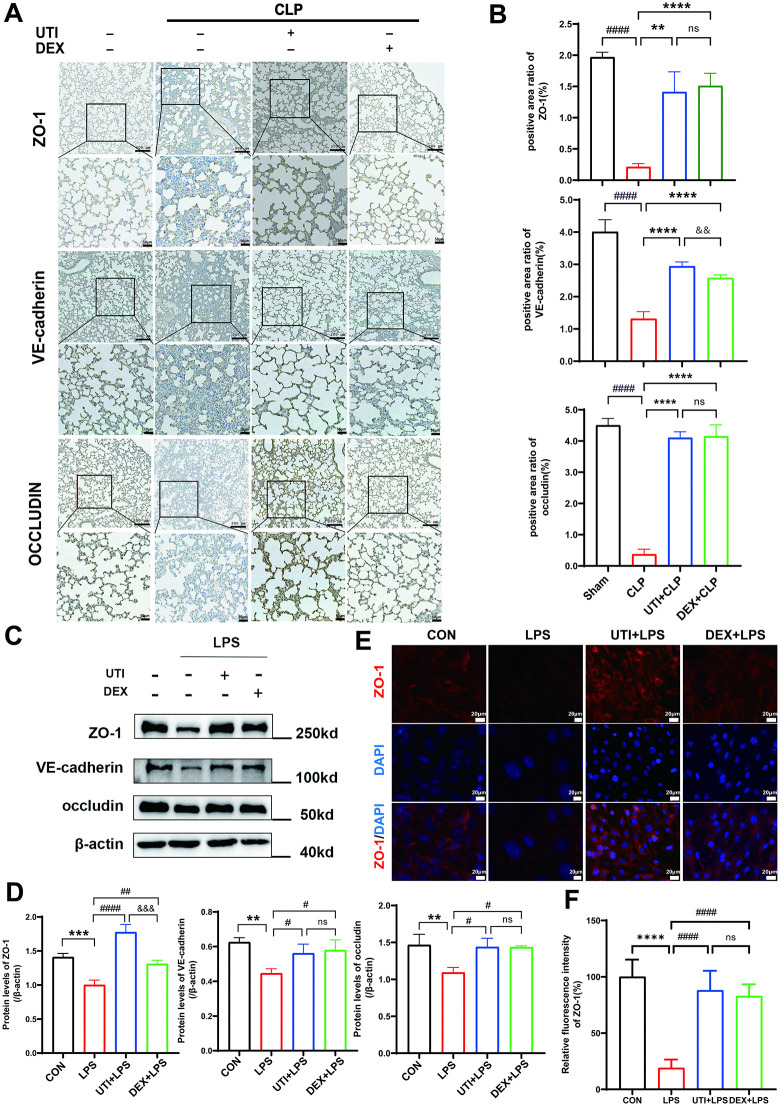
UTI improved endothelial barrier function by protecting the junction proteins. **(A)** Representative immunohistochemical staining images of ZO-1, VE-cadherin, and occludin in lung paraffin sections. Dark brown dots indicate positively stained cells (upper scale bar = 200μm, bottom panel = 50μm). **(B)** Morphometric analysis of immunostained area for each protein in relation to total area was performed quantitatively using Fiji software (n = 6). **(C-D)** Protein levels of ZO-1, VE-cadherin, and occludin in HUVECs were determined by western blot. The relative protein levels are expressed as the ratio of the target protein to Beta-Actin, determined in panel **D. (E)** Immunofluorescent staining showing the expression and distribution of intercellular junction protein (ZO-1) in LPS-challenged HUVEC. RED represents ZO-1, and blue indicates nuclei (Scale bar = 20μm). **(F)** Quantitative analysis of relative fluorescence intensity for ZO-1 was performed using Fiji software (n = 6). All data were expressed as the mean  ±  SD. ns = no significance, ^*^p  <  0.05, ^**^p  <  0.01, ^***^p  <  0.001, and ^****^p  <  0.0001. ^#^*p*  <  0.05, ^##^*p*  <  0.01, ^###^*p*  <  0.001, and ^####^*p* ＜ 0.0001, ^&&&^*p*  <  0.001.

In sepsis, tight junctions (occludin, ZO-1) and adherens junctions (VE-cadherin) are important components in maintaining endothelial barrier integrity and regulating permeability. The immunohistochemistry in lung tissues indicated that protein expression of ZO-1, VE-cadherin, and occludin was markedly downregulated after CLP. UTI or DEX treatment significantly restored the protein abundance (Fig 2A, 2B). Western Blotting (WB) results showed that UTI or DEX significantly restored the expression of junction proteins ZO-1, occludin, and VE-cadherin compared to the LPS group in HUVECs (Fig 2C, 2D). The results of immunofluorescence in HUVECs showed that the expression of ZO-1 was greater in UTI and DEX-treated groups than in the LPS group (Fig 2E, 2F). Above all, these findings indicated that UTI protected the endothelial barrier function by improving the junction proteins.

### The effects of UTI on the expression of angiopoietin-2 (Ang-2) and activation of the phosphatidylinositol 3-kinase (PI3K)/protein kinase B (PKB/AKT)/Forkhead box protein O1(FoxO1) signaling pathway

**Fig 3:**
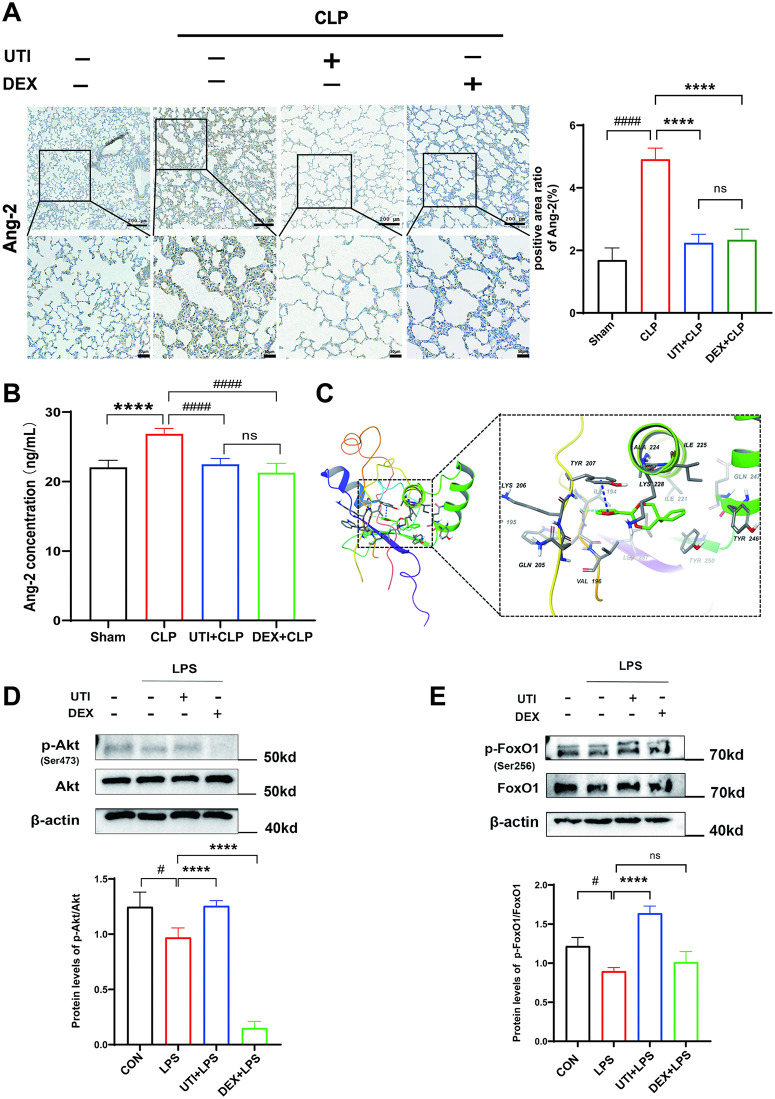
UTI suppressed the expression of angiopoietin-2 (Ang-2) in vivo and activated the PI3K/Akt/FoxO1 signaling pathway in vitro. **(A)** Representative immunohistochemical staining images of Ang-2 in lung paraffin sections. Dark brown dots indicate positively stained cells. Morphometric analysis of immunostained area for each protein in relation to the total area was performed quantitatively using Fiji software (upper scale bar = 200μm, bottom panel = 50μm) (n = 6). **(B)** The levels of Ang-2 in serum were measured by ELISA (n = 6). **(C)** Molecular docking showing the interaction between UTI and phosphatidylinositol 3-kinase (PI3K). **(D-E)** Protein levels of p-Akt and p-FoxO1 in HUVECs were determined by western blot (n  =  3). All data were expressed as the mean  ±  SD. ns = no significance, ^***^p  <  0.0001. ^#^*p*  <  0.05 and ^####^*p* ＜ 0.0001.

Given that Ang-2 initiated a positive feedback loop through restoring FoxO1-driven expression of itself, leading to microvascular leak during infection [11]. However, it’s unclear whether UTI modulates the feedback loop in sepsis. To address this, we next evaluated Ang-2 expression levels in serum and lung tissues. Treatment with UTI or DEX decreased the CLP-induced accumulation levels of Ang-2 in serum. Immunohistochemical analysis indicated that a remarkable reduction of Ang-2 expression by UTI or DEX compared with the CLP rats (Fig 3A, 3B). Interestingly, DEX showed similar effects with UTI on the expression of Ang-2 in CLP rats.

In the context of sepsis, the PI3K/Akt signaling pathway is crucial for maintaining vascular homeostasis in endothelial cells. Because activated PI3K/Akt leads to phosphorylation and nuclear export of FoxO1, inhibiting FoxO1- dependent Ang-2 expression, which maintains endothelial homeostasis [23]. We next examined whether UTI activates PI3K/Akt/FoxO1 signaling pathway and suppresses Ang-2 upregulation during sepsis. To address this, we first performed molecular docking of UTI with PI3K using the Schrödinger suite (Fig 3C). Docking suggested that the ligand’s hydrophobic/aromatic core is encapsulated by a hydrophobic cage formed by residues [ILE194, VAL196, ILE221, Ala224, ILE225, LEU287], which effectively excludes local solvent and reinforces van der Waals and π-interactions. In addition, aromatic interactions with TYR207/TRP195 are observed. The top-ranked pose shows a docking score of −7.47 (scoring units, with more negative scores denoting a better fit), indicating a stable interaction and favorable binding affinity between UTI and PI3K.

**Fig 4:**
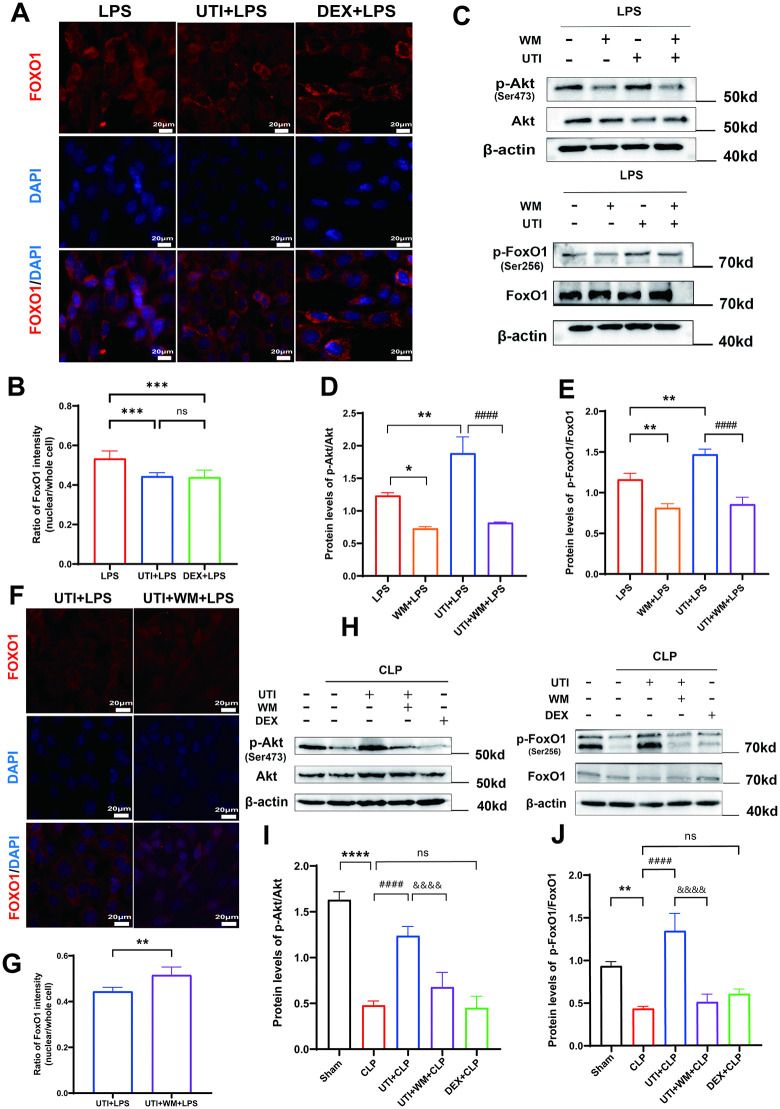
The UTI-induced activation of the PI3K/AKT/FoxO1 pathway was abrogated by wortmannin. **(A-B)** HUVECs were pretreated with UTI (10 KU/mL) or DEX (10 μM) for 1 h before LPS stimuli for 24 **h.** Immunofluorescent staining of FoxO1 showing the nuclear translocation in LPS-challenged HUVECs with UTI or DEX treatment (Scale bar = 20μm). Quantitative analysis of the ratio of nuclear area to total area in FoxO1 protein immunofluorescence staining regions was performed using Fiji software in panel B (n = 6). **(C-E)** HUVECs were pretreated with UTI (10 KU/mL), with or without the PI3K inhibitor wortmannin (WM, 1 μM). Protein levels of p-Akt and p-FoxO1 in HUVECs were analyzed by western blot. Quantitative data of Akt and FoxO1 phosphorylation in HUVECs in panels D, E (n = 3). **(F-G)** Immunofluorescent staining of FoxO1 showing the nuclear translocation in HUVECs (Scale bar = 20μm). Quantitative analysis of the ratio of nuclear area to total area in FoxO1 immunofluorescence staining regions was performed using Fiji software in panel G (n = 6). **(H-J)** CLP rats were intraperitoneally injected with UTI or WM (0.7 mg/kg). Representative western blots of p-Akt and p-FoxO1 in the lung tissue harvested 24 h after CLP surgery. Quantitative data of Akt and FoxO1 phosphorylation in the lung tissues in panel J (n = 3). All data were expressed as the mean  ±  SD. ns = no significance, ^*^p  <  0.05, ^**^p  <  0.01, ^***^p  <  0.001, and ^****^p  <  0.0001. ^##^*p*  <  0.01 and ^####^*p* ＜ 0.0001. ^&&&&^*p*  <  0.0001.

With the administration of UTI, phosphorylated Akt and FoxO1 were upregulated compared with LPS stimulation in HUVECs (Fig 3D, 3E). However, the phosphorylation of FoxO1 was not altered in the DEX group compared to the LPS group. In addition, immunofluorescent staining showed that UTI led to the nuclear export of FoxO1. It also observed that DEX results in the nuclear export of FoxO1, which seems contradictory to the unaffected expression level of p-FoxO1 (Fig 4A, 4B). Existing studies on skeletal muscle have shown that dexamethasone activates FoxO1 nuclear export via p300-dependent acetylation [24]. Collectively, it implied that dexamethasone may regulate FoxO1 acetylation in endothelial cells during sepsis.

We next explored whether UTI activates AKT and FoxO1 through PI3K. WB results confirmed that the PI3K inhibitor wortmannin (1 μM) suppressed the UTI-induced promotion of AKT and FoxO1 phosphorylation in the LPS-induced HUVECs (Fig 4C, 4E). The result of immunofluorescence of HUVUCs showed that wortmannin retained FoxO1 in the nucleus (Fig 4F, 4G). In CLP rats, the results of WB also revealed that wortmannin (0.7 mg/kg) prevented the phosphorylation of Akt and FoxO1 (Fig 4H, 4J). The results indicated that UTI reduced the expression of Ang-2 and activated the PI3K/Akt/FoxO1 signaling pathway.

### The protective effect of UTI on SALI and the endothelial barrier is mediated by the PI3K/Akt/FoxO1 pathway

**Fig 5:**
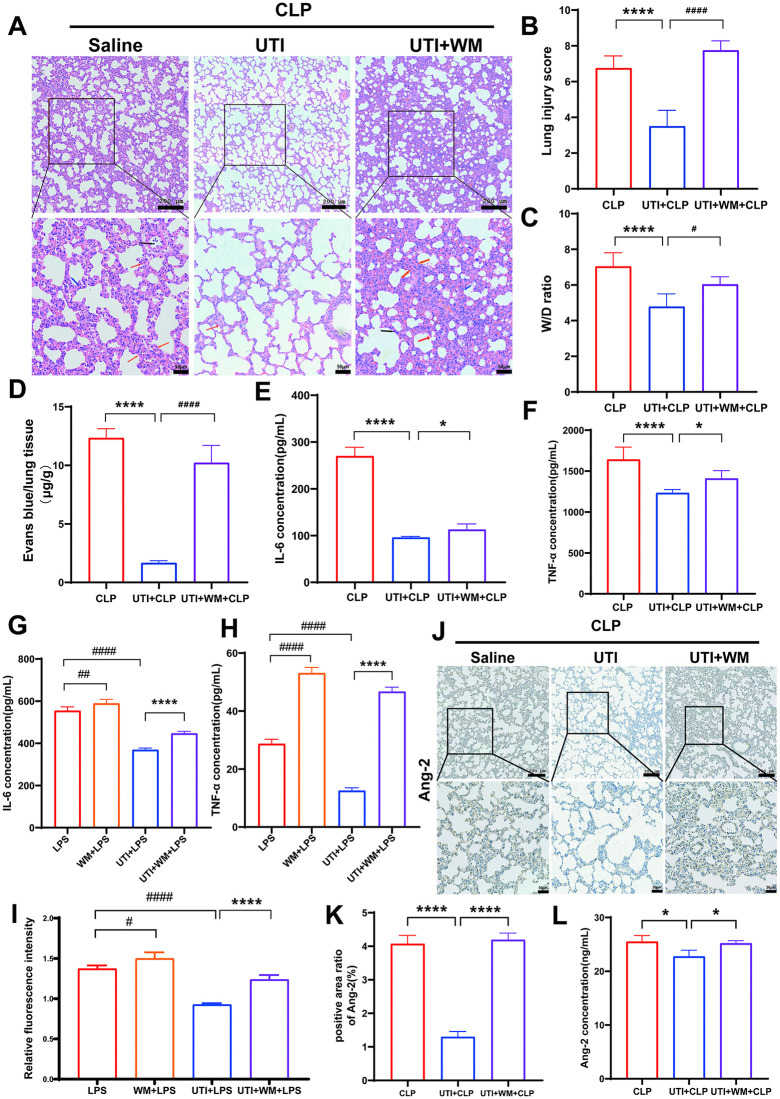
The protective effect of UTI in SALI and endothelial dysfunction is mediated by the PI3K/Akt/FoxO1 pathway. **(A-B)** CLP model rats were intraperitoneally injected with UTI or WM. H&E staining showing the tissue injury in the lung (upper scale bar = 200μm, bottom panel = 50μm). Quantification of lung injury scores was determined in panel B (n = 6). **(C-D)** Lung wet-to-dry weight ratio and Evans Blue dye extravasation were determined in lung tissues (n = 6). **(E-F)** The levels of TNF-α and IL-6 in serum were measured by ELISA (n = 6). **(G-H)** HUVECs were pretreated with UTI or WM before LPS stimuli for 1 hour. Levels of IL-6 and TNF-α in the supernatants of HUVECs were examined by ELISA (n = 6). **(I)** The fluorescence signal intensity of FITC-FD40 in HUVECs (n = 6). **(J-K)** Representative immunohistochemical staining images and relative analysis of Ang-2 in lung paraffin sections (upper scale bar = 200μm, bottom panel = 50μm) (n = 6). **(L)** The levels of Ang-2 in serum were measured by ELISA (n = 6). All data were expressed as the mean  ±  SD. ns = no significance, ^*^p  <  0.05 and ^****^p  <  0.0001. ^*#*^*p  <*  0.01, ^*##*^*p*  <  0.01, and ^*####*^*p* ＜ 0.0001.

We used the PI3K inhibitor (wortmannin) to confirm whether the protective effects of UTI on SALI and the endothelial barrier are mediated by the PI3K/Akt/FoxO1 pathway. In CLP rats, the severity of SALI was found to be significantly greater in the wortmannin and UTI treatment group than that in the UTI alone group (Fig 5A, 5D). Also, the administration of wortmannin reversed the reduction of UTI on inflammatory factors in vivo and in vitro (Fig 5E, 5H). Moreover, wortmannin eliminated the UTI-induced reduction of the fluorescence signal intensity of FITC-FD40 in vitro (Fig 5I). Furthermore, the results showed that wortmannin abolished the reduction of Ang-2 induced by UTI in both septic serum and lung tissues (Fig 5J, 5L).

**Fig 6:**
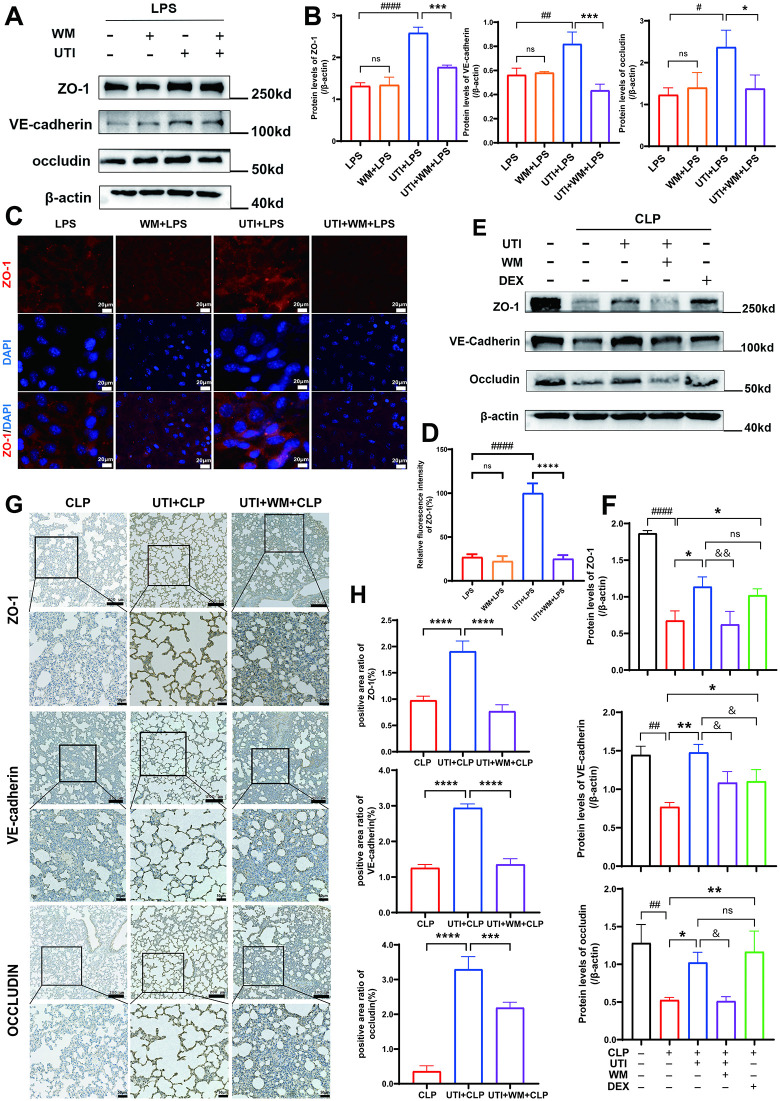
The protective effect of UTI on junction proteins is mediated by the PI3K/Akt/FoxO1 pathway. **(A-B)** Protein levels of ZO-1, VE-cadherin, and occludin in HUVECs were analyzed by western blot. The relative protein levels are expressed in panel B (n = 3). **(C)** Representative immunofluorescence staining images of ZO-1 in HUVECs (n = 6). (Scale bar = 20μm). **(D)** Quantitative analysis of relative fluorescence intensity for ZO-1 was performed using Fiji software (n = 6). **(E-F)** Representative Western blots of ZO-1, VE-cadherin, and occludin protein expression in rat lung tissues. The relative protein levels are expressed in panel E (n = 3). **(G-H)** Immunohistochemical staining showing the levels and distribution of ZO-1, VE-cadherin, and occludin in lung tissues in panel G (upper scale bar = 200μm, bottom panel = 50μm). All data were expressed as the mean  ±  SD. ns = no significance, ^*^p  <  0.05, ^**^p  <  0.01, ^***^p  <  0.001 and ^****^p  <  0.0001. ^*##*^*p*  <  0.01. ^*&*^*p*  <  0.01, ^*&&*^*p*  <  0.01.

Subsequently, the impacts of wortmannin on endothelial junction proteins were verified by WB, immunofluorescence, and immunohistochemical analysis. The results of WB showed that the upregulation of junction proteins treated by UTI was inhibited upon the intervention of wortmannin in LPS-induced HUVECs (Fig 6A, 6B). Immunofluorescence staining of HUVECs further confirmed that wortmannin inhibited the protective effect of UTI on ZO-1 (Fig 6C, 6D). In CLP rats, the WB results indicated that wortmannin abolished the protective effects of UTI on junction proteins (Fig 6E, 6F), which was also confirmed through the immunohistochemical analysis (Fig 6G, 6H). Altogether, these results demonstrated that UTI prevented SALI and endothelial dysfunction through activating the PI3K/Akt/FoxO1 pathway and inhibiting the expression of Ang-2.

## Discussion

Our study provides compelling evidence that Ulinastatin (UTI) ameliorates sepsis-associated acute lung injury (SALI) primarily by preserving endothelial barrier integrity and attenuating capillary leakage. We identified a novel molecular mechanism underlying this protection: UTI binds to PI3K, activating the PI3K/Akt/FoxO1 signaling axis, which subsequently disrupts a critical positive feedback loop driven by the transcription factor FoxO1 that sustains high levels of angiopoietin-2 (Ang-2). This mechanistic insight significantly advances our understanding of UTI#39;s therapeutic actions beyond its known anti-inflammatory properties. More importantly, we identified a novel mechanism underlying this protection, whereby UTI activates the PI3K/Akt/FoxO1 signaling axis and disrupts the positive feedback loop that sustains Ang-2 overexpression and endothelial barrier dysfunction. These findings extend the current understanding of UTI beyond its general anti-inflammatory properties and provide mechanistic insight into its role in maintaining endothelial homeostasis during sepsis.

Sepsis is closely related to the microcirculatory dysfunction [25]. Endothelial cells are considered vital mediators of septic shock and play a crucial role in maintaining barrier integrity across multiple organs and tissues [26]. UTI purified from fresh human urine is a broad-spectrum protease inhibitor used widely in pancreatitis and critical illnesses for several decades. In LPS-induced ARDS mice, UTI alleviated pulmonary edema by inhibiting the pulmonary endothelial glycocalyx shedding [27] and suppressing ferroptosis by downregulating KEAP1 and activating Nrf2 [28]. In sepsis-associated acute kidney injury (SAKI), UTI improved both cortical and medullary perfusion by maintaining VE-cadherin expression [29,30]. A recent clinical study showed that UTI improved therapeutic efficacy in children with septic shock by reducing inflammatory markers [31]. Our study provides deeper insights into the impact of UTI on capillary endothelial function, which positively influenced the restoration of intercellular junction proteins and the maintenance of endothelial homeostasis.

FoxO1, a member of the forkhead transcription factor family, is involved in processes such as DNA repair, cell cycle control, stress resistance, and cell metabolism [32]. Our data robustly show that UTI promotes FoxO1 phosphorylation and its export from the nucleus. This is functionally critical because nuclear FoxO1 is a potent driver of Ang-2 gene expression [11,13]. The consequent downregulation of Ang-2 by UTI is therefore a direct outcome of interrupted FoxO1 signaling. Furthermore, FoxO1 was the key downstream effector of the PI3K/Akt signaling pathway, which was negatively regulated by Akt-mediated phosphorylation and nuclear exclusion [33]. The PI3K/Akt pathway is a cornerstone of endothelial survival and quiescence. We demonstrated that UTI directly engages with PI3K, as supported by molecular docking, and initiates a signaling cascade leading to Akt phosphorylation. The fact that the PI3K inhibitor wortmannin completely abrogated the benefits of UTI—reversing its effects on FoxO1 phosphorylation, Ang-2 suppression, junction protein preservation, and barrier protection—firmly establishes the PI3K/Akt/FoxO1 pathway as the primary conduit for UTI#39;s action in SALI.

Furthermore, our data confirm that UTI robustly preserves key endothelial junctional proteins, namely ZO-1, occludin, and VE-cadherin. The loss of these proteins is a hallmark of barrier failure in sepsis. It is noteworthy that these proteins can be shed into the circulation in soluble forms (e.g., soluble VE-cadherin [34]), serving as biomarkers of endothelial damage. While our study focused on total cellular protein levels, it raises the intriguing possibility that UTI may also reduce the proteolytic shedding of these junctions, potentially through inhibition of sheddases like ADAM10 [35]. This potential secondary mechanism warrants further investigation.

The observed effect of dexamethasone on FoxO1 localization, without altering its phosphorylation state, hints at alternative regulatory mechanisms such as acetylation [24] and underscores that UTI and corticosteroids, while potentially converging on similar endpoints, operate through distinct molecular routes. Above all, the comparable efficacy between UTI and dexamethasone in our models highlights the therapeutic potential of UTI. Given the ongoing controversies [36] and significant adverse effects associated with chronic corticosteroid use [37], UTI presents itself as a promising steroid-sparing agent or a complementary therapeutic for sepsis management.

Ang-2 has emerged as a pivotal mediator of sepsis pathology, and its elevated levels are a harbinger of poor outcomes [9,38]. By destabilizing the vasculature, Ang-2 initiates a self-perpetuating cycle of leakage and inflammation. Our discovery that UTI effectively silences this cycle by targeting its transcriptional origin provides a powerful upstream intervention strategy. This offers a plausible explanation for the clinical efficacy of UTI in septic shock [31]. Recent studies exploring therapeutic plasma exchange (TPE) have shown that rapid reduction of circulating Ang-2 can improve survival in severe sepsis [39,40]. While effective, TPE is invasive and non-specific. UTI, with its more targeted pharmacological action and favorable safety profile, could represent an attractive alternative or even a maintenance therapy following initial TPE for sustained endothelial protection.

**Fig 7:**
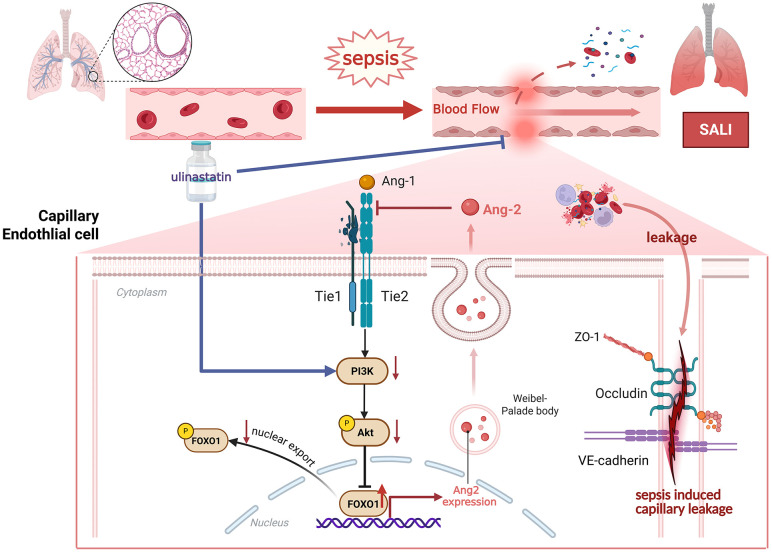
Graphical abstract. In sepsis, Ang-2 was activated for endothelial destabilization, leading to endothelial barrier dysfunction. UTI attenuated capillary leakage in sepsis-associated acute lung injury by activating the PI3K/Akt/FoxO1 pathway and breaking the vicious cycle of FoxO1-dependent Ang-2 production. Created in BioRender. Yating, **L.** (2026) https://BioRender.com/wud3kqb.

In conclusion, our study elucidates a precise mechanistic pathway through which UTI confers protection against SALI. By activating the PI3K/Akt/FoxO1 axis, UTI halts the FoxO1- dependent overproduction of Ang-2, thereby breaking a key vicious cycle in septic endothelial dysfunction and stabilizing the vascular barrier (Fig 7). These findings not only deepen our understanding of UTI’s pharmacology but also solidify its rationale as a targeted therapeutic agent for mitigating capillary leakage in sepsis.

## Supporting information

S1 File Original result images include Western blot images. (PDF)

S2 File Ethical Approval for Research Involving Animals. (PDF)

## References

[1] Fleischmann-Struzek C, Rudd K. Challenges of assessing the burden of sepsis. Med Klin Intensivmed Notfmed. 2023;118(Suppl 2):68–74. doi: 10.1007/s00063-023-01088-7 37975898 PMC10733211

[2] Rudd KE, Johnson SC, Agesa KM, Shackelford KA, Tsoi D, Kievlan DR, et al. Global, regional, and national sepsis incidence and mortality, 1990-2017: analysis for the Global Burden of Disease Study. Lancet. 2020;395(10219):200–11. 31954465 10.1016/S0140-6736(19)32989-7 PMC6970225

[3] Saravi B, Goebel U, Hassenzahl LO, Jung C, David S, Feldheiser A, et al. Capillary leak and endothelial permeability in critically ill patients: a current overview. Intensive Care Med Exp. 2023;11(1):96. doi: 10.1186/s40635-023-00582-8 38117435 PMC10733291

[4] McMullan RR, McAuley DF, O’Kane CM, Silversides JA. Vascular leak in sepsis: physiological basis and potential therapeutic advances. Crit Care. 2024;28(1):97. doi: 10.1186/s13054-024-04875-6 38521954 PMC10961003

[5] Maniatis NA, Orfanos SE. The endothelium in acute lung injury/acute respiratory distress syndrome. Curr Opin Crit Care. 2008;14(1):22–30. doi: 10.1097/MCC.0b013e3282f269b9 18195622

[6] Tang F, Zhao X-L, Xu L-Y, Zhang J-N, Ao H, Peng C. Endothelial dysfunction: Pathophysiology and therapeutic targets for sepsis-induced multiple organ dysfunction syndrome. Biomed Pharmacother. 2024;178:117180. doi: 10.1016/j.biopha.2024.117180 39068853

[7] Jiang J, Huang K, Xu S, Garcia JGN, Wang C, Cai H. Targeting NOX4 alleviates sepsis-induced acute lung injury via attenuation of redox-sensitive activation of CaMKII/ERK1/2/MLCK and endothelial cell barrier dysfunction. Redox Biol. 2020;36:101638. doi: 10.1016/j.redox.2020.101638 32863203 PMC7381685

[8] Qiao X, Yin J, Zheng Z, Li L, Feng X. Endothelial cell dynamics in sepsis-induced acute lung injury and acute respiratory distress syndrome: pathogenesis and therapeutic implications. Cell Commun Signal. 2024;22(1):241. doi: 10.1186/s12964-024-01620-y 38664775 PMC11046830

[9] Lymperopoulou K, Velissaris D, Kotsaki A, Antypa E, Georgiadou S, Tsaganos T, et al. Angiopoietin-2 associations with the underlying infection and sepsis severity. Cytokine. 2015;73(1):163–8. doi: 10.1016/j.cyto.2015.01.022 25748839

[10] Richter RP, Ashtekar AR, Zheng L, Pretorius D, Kaushlendra T, Sanderson RD, et al. Glycocalyx heparan sulfate cleavage promotes endothelial cell angiopoietin-2 expression by impairing shear stress-related AMPK/FoxO1 signaling. JCI Insight. 2022;7(15):e155010. doi: 10.1172/jci.insight.155010 35763350 PMC9462499

[11] Kim M, Allen B, Korhonen EA, Nitschké M, Yang HW, Baluk P, et al. Opposing actions of angiopoietin-2 on Tie2 signaling and FOXO1 activation. J Clin Invest. 2016;126(9):3511–25. doi: 10.1172/JCI84871 27548529 PMC5004955

[12] Daly C, Wong V, Burova E, Wei Y, Zabski S, Griffiths J, et al. Angiopoietin-1 modulates endothelial cell function and gene expression via the transcription factor FKHR (FOXO1). Genes Dev. 2004;18(9):1060–71. doi: 10.1101/gad.1189704 15132996 PMC406295

[13] Akwii RG, Sajib MS, Zahra FT, Mikelis CM. Role of Angiopoietin-2 in Vascular Physiology and Pathophysiology. Cells. 2019;8(5):471. doi: 10.3390/cells8050471 31108880 PMC6562915

[14] Ji M, Chen T, Wang B, Chen M, Ding Q, Chen L, et al. Effects of ulinastatin combined with mechanical ventilation on oxygen metabolism, inflammation and stress response and antioxidant capacity of ARDS. Exp Ther Med. 2018;15(6):4665–70. doi: 10.3892/etm.2018.6012 29805484 PMC5952097

[15] Tanaka R, Fujita M, Tsuruta R, Fujimoto K, Aki HS, Kumagai K, et al. Urinary trypsin inhibitor suppresses excessive generation of superoxide anion radical, systemic inflammation, oxidative stress, and endothelial injury in endotoxemic rats. Inflamm Res. 2010;59(8):597–606. doi: 10.1007/s00011-010-0166-8 20148283

[16] Wang R, Song W, Xie C, Zhong W, Xu H, Zhou Q, et al. Urinary Trypsin Inhibitor Protects Tight Junctions of Septic Pulmonary Capillary Endothelial Cells by Regulating the Functions of Macrophages. J Inflamm Res. 2021;14:1973–89. doi: 10.2147/JIR.S303577 34045879 PMC8149216

[17] Fang M, Zhong W-H, Song W-I, Deng Y-Y, Yang D-M, Xiong B. Ulinastatin ameliorates pulmonary capillary endothelial permeability induced by sepsis through protection of tight junctions via inhibition of TNF-α and related pathways. Frontiers in Pharmacology. 2018;9. 10.3389/fphar.2018.00823 PMC6099086 30150933

[18] Jiang Y-X, Huang Z-W. Ulinastatin alleviates pulmonary edema by reducing pulmonary permeability and stimulating alveolar fluid clearance in a rat model of acute lung injury. Iran J Basic Med Sci. 2022;25(8):1002–8. doi: 10.22038/IJBMS.2022.64655.14230 36159332 PMC9464339

[19] Qiao J, Guo S, Huang X, Zhang L, Li F, Fan Y. Expression of Angiopoietin-2 in Lung Tissue of Juvenile SD Rats with Lipopolysaccharide-Induced Acute Lung Injury and the Role of Ulinastatin. Arch Immunol Ther Exp (Warsz). 2023;71(1):23. doi: 10.1007/s00005-023-00688-7 37882869

[20] Lu Y-N, Wang X-H, Xu B-C, Zheng Y-F, Zhang X, Liu Y, et al. Urinary trypsin inhibitor exerts multifaceted regulation of angiopoietin 2 in septic cardiomyopathy. Eur J Pharmacol. 2025;1003:177944. doi: 10.1016/j.ejphar.2025.177944 40651788

[21] Li X, Yang C, Gulifeire T, Wang Y, Yu X. Ulinastatin protects intestinal mucosal barrier by inhibiting the activation of intestinal NLRP3 inflammasomes in septic rats. Zhonghua Wei Zhong Bing Ji Jiu Yi Xue. 2021;33(2):192–7. doi: 10.3760/cma.j.cn121430-20201208-00747 33729139

[22] Jain D, Atochina-Vasserman E, Kadire H, Tomer Y, Inch A, Scott P, et al. SP-D-deficient mice are resistant to hyperoxia. Am J Physiol Lung Cell Mol Physiol. 2007;292(4):L861–71. doi: 10.1152/ajplung.00145.2006 17158597

[23] Cong X, Kong W. Endothelial tight junctions and their regulatory signaling pathways in vascular homeostasis and disease. Cell Signal. 2020;66:109485. doi: 10.1016/j.cellsig.2019.109485 31770579

[24] Chamberlain W, Gonnella P, Alamdari N, Aversa Z, Hasselgren P-O. Multiple muscle wasting-related transcription factors are acetylated in dexamethasone-treated muscle cells. Biochem Cell Biol. 2012;90(2):200–8. doi: 10.1139/o11-082 22292478

[25] McMullan RR, McAuley DF, O’Kane CM, Silversides JA. Vascular leak in sepsis: physiological basis and potential therapeutic advances. Crit Care. 2024;28(1):97. doi: 10.1186/s13054-024-04875-6 38521954 PMC10961003

[26] Tang F, Zhao X-L, Xu L-Y, Zhang J-N, Ao H, Peng C. Endothelial dysfunction: Pathophysiology and therapeutic targets for sepsis-induced multiple organ dysfunction syndrome. Biomed Pharmacother. 2024;178:117180. doi: 10.1016/j.biopha.2024.117180 39068853

[27] Wang L, Huang X, Kong G, Xu H, Li J, Hao D, et al. Ulinastatin attenuates pulmonary endothelial glycocalyx damage and inhibits endothelial heparanase activity in LPS-induced ARDS. Biochem Biophys Res Commun. 2016;478(2):669–75. doi: 10.1016/j.bbrc.2016.08.005 27498004

[28] Guo Q, Gao X, Ren J, Deng G, Li R, Zhang C, et al. Inhibition of ferroptosis by serine protease inhibitor attenuates acute respiratory distress syndrome. Arch Biochem Biophys. 2025;773:110596. doi: 10.1016/j.abb.2025.110596 40849045

[29] Li T, Ji X, Liu J, Guo X, Pang R, Zhuang H, et al. Ulinastatin Improves Renal Microcirculation by Protecting Endothelial Cells and Inhibiting Autophagy in a Septic Rat Model. Kidney Blood Press Res. 2022;47(4):256–69. doi: 10.1159/000521648 35016182

[30] Yang X-Y, Song J, Hou S-K, Fan H-J, Lv Q, Liu Z-Q, et al. Ulinastatin ameliorates acute kidney injury induced by crush syndrome inflammation by modulating Th17/Treg cells. Int Immunopharmacol. 2020;81:106265. doi: 10.1016/j.intimp.2020.106265 32044661

[31] Gao S, Cheng S, Zhang Q, Cheng Z, Chen L, Ren Z, et al. Effects of ulinastatin on therapeutic outcomes and inflammatory markers in pediatric septic shock patients. Sci Rep. 2025;15(1):16624. doi: 10.1038/s41598-025-00629-8 40360546 PMC12075584

[32] Szydłowski M, Jabłońska E, Juszczyński P. FOXO1 transcription factor: a critical effector of the PI3K-AKT axis in B-cell development. Int Rev Immunol. 2014;33(2):146–57. doi: 10.3109/08830185.2014.885022 24552152

[33] Brunet A, Bonni A, Zigmond MJ, Lin MZ, Juo P, Hu LS, et al. Akt promotes cell survival by phosphorylating and inhibiting a Forkhead transcription factor. Cell. 1999;96(6):857–68. doi: 10.1016/s0092-8674(00)80595-4 10102273

[34] Flemming S, Burkard N, Renschler M, Vielmuth F, Meir M, Schick MA, et al. Soluble VE-cadherin is involved in endothelial barrier breakdown in systemic inflammation and sepsis. Cardiovasc Res. 2015;107(1):32–44. doi: 10.1093/cvr/cvv144 25975259

[35] Schulz B, Pruessmeyer J, Maretzky T, Ludwig A, Blobel CP, Saftig P, et al. ADAM10 regulates endothelial permeability and T-Cell transmigration by proteolysis of vascular endothelial cadherin. Circ Res. 2008;102(10):1192–201. doi: 10.1161/CIRCRESAHA.107.169805 18420943 PMC2818019

[36] Annane D, Briegel J, Seymour CW. Corticosteroids in sepsis: certainties and shadows. Intensive Care Med. 2025;51(10):1890–3. doi: 10.1007/s00134-025-08044-3 40699324

[37] Chaudhuri D, Israelian L, Putowski Z, Prakash J, Pitre T, Nei AM, et al. Adverse Effects Related to Corticosteroid Use in Sepsis, Acute Respiratory Distress Syndrome, and Community-Acquired Pneumonia: A Systematic Review and Meta-Analysis. Crit Care Explor. 2024;6(4):e1071. doi: 10.1097/CCE.0000000000001071 38567382 PMC10986917

[38] Scholz A, Plate KH, Reiss Y. Angiopoietin-2: a multifaceted cytokine that functions in both angiogenesis and inflammation. Ann N Y Acad Sci. 2015;1347:45–51. doi: 10.1111/nyas.12726 25773744

[39] Stahl K, Wand P, Seeliger B, Wendel-Garcia PD, Schmidt JJ, Schmidt BMW, et al. Clinical and biochemical endpoints and predictors of response to plasma exchange in septic shock: results from a randomized controlled trial. Crit Care. 2022;26(1):134. doi: 10.1186/s13054-022-04003-2 35551628 PMC9097091

[40] Keith PD, Wells AH, Hodges J, Fast SH, Adams A, Scott LK. The therapeutic efficacy of adjunct therapeutic plasma exchange for septic shock with multiple organ failure: a single-center experience. Crit Care. 2020;24(1):518. doi: 10.1186/s13054-020-03241-6 32831133 PMC7443810

