# Supplementary figures and images for: Ulinastatin attenuates capillary leakage and suppresses FoxO1-dependent angiopoietin-2 in sepsis-associated acute lung injury via PI3K pathway
Source: PLoS One. 2026 Apr 28;21(4):e0348261. doi: 10.1371/journal.pone.0348261 (PMC13123937; doi:10.1371/journal.pone.0348261)

Figure 2C

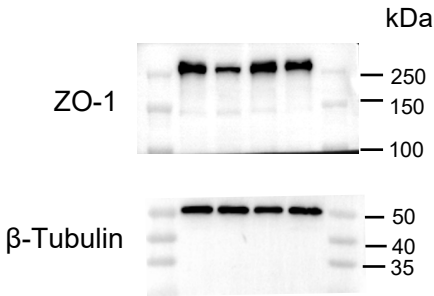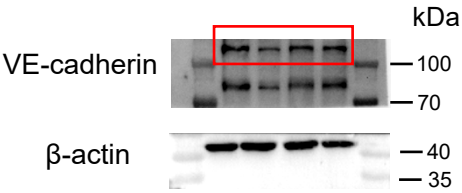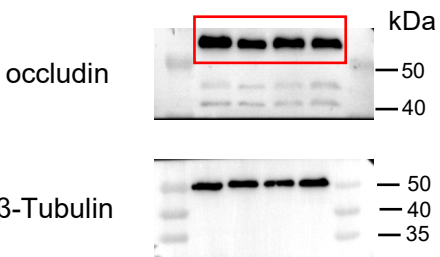

Figure 3D

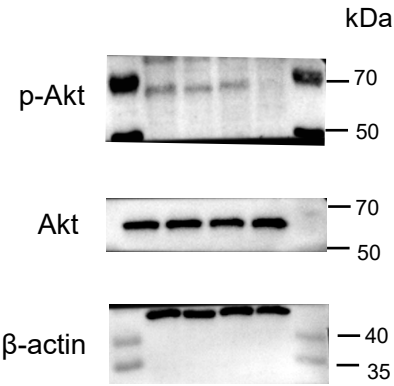

Figure 3E

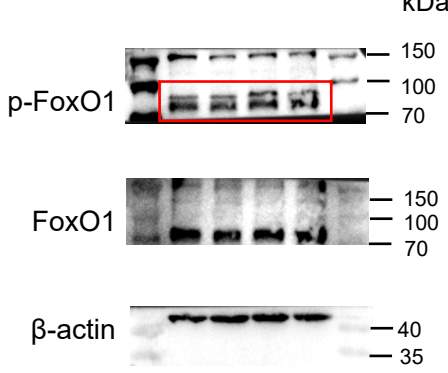

Figure 4C

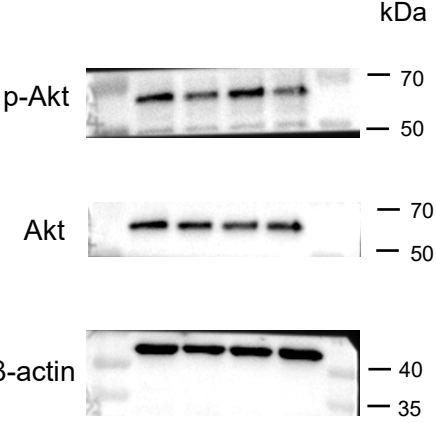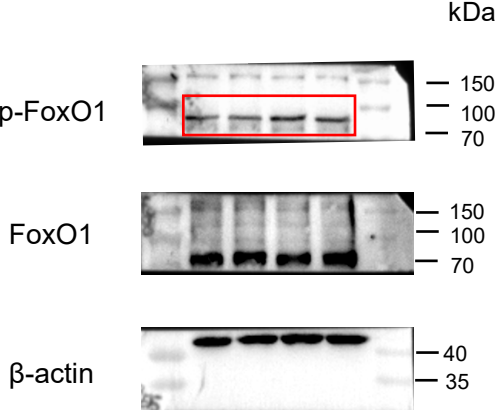

Figure 4H

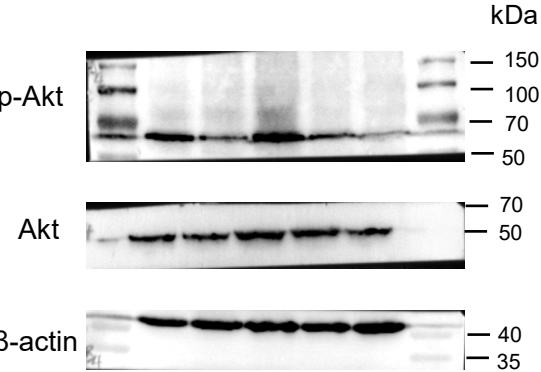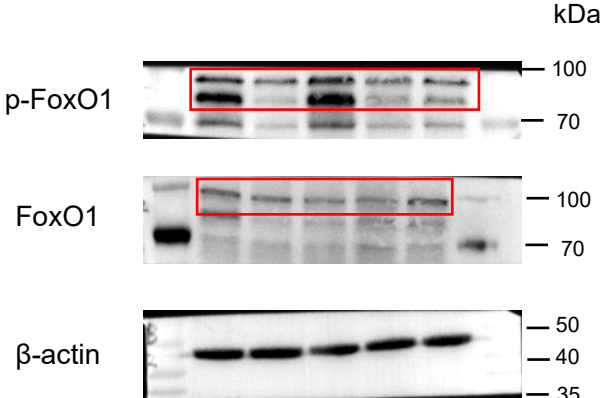

Figure 6A

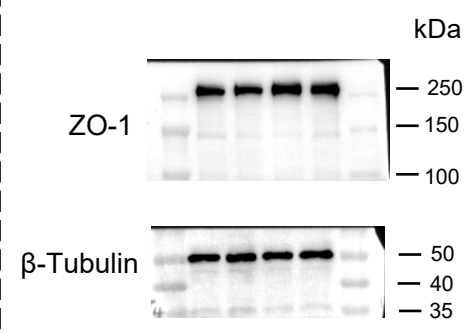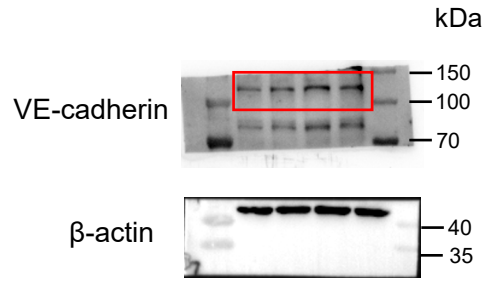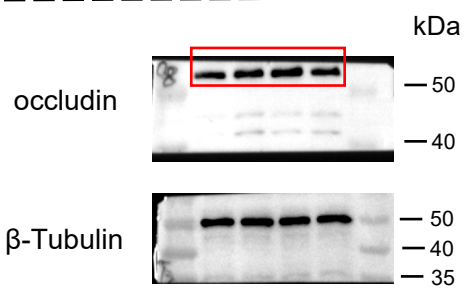

Figure 6D

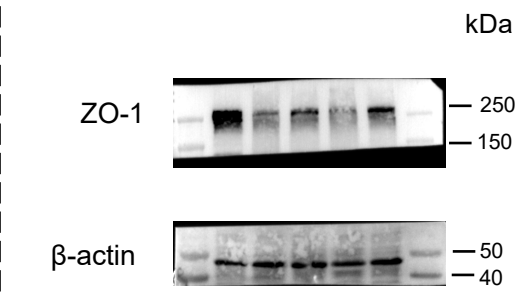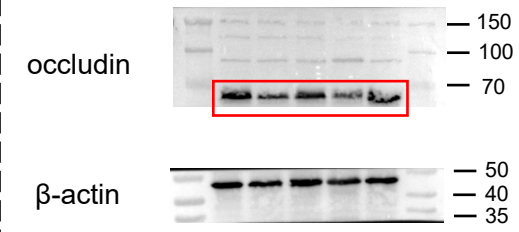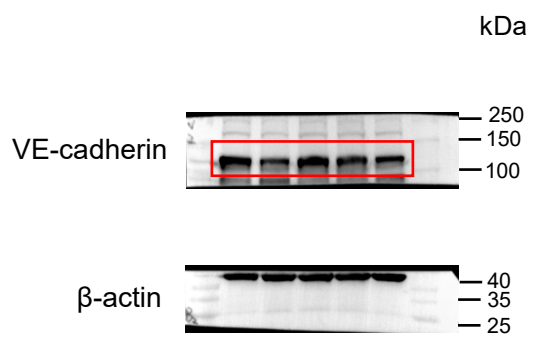

Supplement: S1 File — (PDF) [file pone.0348261.s001.pdf]
