# Supplementary material for: Ulinastatin attenuates capillary leakage and suppresses FoxO1-dependent angiopoietin-2 in sepsis-associated acute lung injury via PI3K pathway
Source: PLoS One. 2026 Apr 28;21(4):e0348261. doi: 10.1371/journal.pone.0348261 (PMC13123937; doi:10.1371/journal.pone.0348261)
Supplement: S2 File — (PDF) [file pone.0348261.s002.pdf]

兰州大学第二医院实验动物福利伦理审查表

Application Format for Ethical Approval for Research Involving Animals of Lanzhou University Second Hospital

申请日期： 2024 年 8 月 22 日　　　　受理编号：D2024-773

Appl. Date　2024 Y 8　M 22　D　　　　Appl. No.

<table>
<tr><td colspan="5">课题名称：乌司他丁通过调控 PI3K/Akt/FOXO1 信号通路影响脂多糖诱导的脓毒症相关毛细血管渗漏综合征
Program</td></tr>
<tr><td colspan="5">项目类型：2024 年度兰州大学第二医院“萃英科技创新”计划
Project Type</td></tr>
<tr><td colspan="3">项目负责人 马莉
Principal Investigator (PI)</td><td colspan="2">单位 兰州大学第二医院
Department</td></tr>
<tr><td colspan="3">职称 主任医师
Professional Title</td><td>联系电话 13893679654
Tel.No.</td><td>邮箱 ery_mali@lzu.edu.cn
Email</td></tr>
<tr><td>项目执行人 Project Executor</td><td colspan="4">马莉</td></tr>
<tr><td>联系电话 Tel.No.</td><td colspan="4">13893679654</td></tr>
<tr><td>邮箱 Email</td><td colspan="4">ery_mali@lzu.edu.cn</td></tr>
<tr><td colspan="5">拟实验时间　2025 年 1 月 1 日至 2027 年 12 月 31 日
Experimental Period　2025 Y 1 M 1 D to 2027 Y 12 M 31 D</td></tr>
<tr><td rowspan="2">实验动物
Laboratory animal</td><td colspan="2">品种/品系 SD 大鼠（日龄：20 天） 白色
Breed / Strain</td><td colspan="2">等级（请在方框内打√）
Grade [illegible] 1.无特定病原体级实验动物 Specific Pathogen Free-SPF□ 2.无菌级实验动物 Germ Free-GF</td></tr>
<tr><td>数量（只）Number</td><td>雌/Female（♀）__;
雄/Male（♂）_60__;</td><td colspan="2">平均体重： __200 - 250 __ g
Average Weight</td></tr>
<tr><td>选择实验动物种类及数量的理由
Reasons for the choice of species and numbers of animals to be used</td><td colspan="4">每组至少 6 只有效例数，因此每组 8 只，实验总共 40 只大鼠，备用 20 只大鼠。</td></tr>
<tr><td>实验动物培训情况介绍
Description of laboratory animal training</td><td colspan="4">项目课题组成员马鑫参加实验动物及动物实验技术培训，并考核合格已获得实验动物技术培训证书。其余课题组成员均学习并掌握实验动物相关理论及基本操作技术。</td></tr>
</table>

动物实验设计
Animal experiment design

实验方案及主要实验操作
Experimental scheme and main operation

动物实施安乐死后进行心脏采血，收集血液后分离血清，用酶联免疫吸附法（ELISA）检测血清炎症因子的水平。将肺泡灌洗液-80℃保存，将肺组织分装分别用固定液固定或液氮保存以便后续进行切片和提取肺组织蛋白 WB 检测 LC3、p-PI3K、PI3K、p-Akt、Akt、mTOR、p-mTOR、FOXO1、p-FOXO1 等相关蛋白表达。

实验分组（分组情况、组数、每组的动物数、实验周期） Grouping experiment (Grouping situation, Number of groups, Number of animals per group, Experimental cycle)

采用随机数表法分为 5 组：对照组，LPS 组，LPS+乌司他丁组（低、中、高剂量），每组至少 6 只有效例数，每组至少 8 只，周期约 10 个月。

实验终点及仁慈终点描述 Humane endpoint or experimental terminative indicator

实验终点：大鼠药物干预建模 24 小时后结束。

仁慈终点：使用 CCAC 和 OECD 推荐使用的疼痛/痛苦等级量化表，评价动物状态和行为，来评估动物所承受的疼痛和痛苦的程度，如评分大于 10 分，则判定终止实验，麻醉后处死实验动物。

麻醉剂名称及剂量 ___腹腔注射___ （3 %戊巴比妥钠）
Name and dose of anesthetic

麻醉方式 Anesthesia methods

- ☐ 灌胃 Intragastric
- ☐ 皮下 Subcutaneous
- ■ 腹腔 Intraperitoneal
- ☐ 静脉 Intravenous
- ☐ 其他 Others

如其他，请注明：
Others, detailed description:

处死方式 Death conduct

- ☐ 麻醉后颈椎脱臼 Cervical dislocation after anesthesia
- ■ 过量麻醉 Anesthesia overdose
- ☐ 二氧化碳吸入 CO_2 suffocated
- ☐ 其他 Others 如其他，请注明：Others, detailed description:

尸体处理方式 Disposal of animals' remains

- ■ 统一送至甘肃省危废中心处理 Gansu Hazardous Waste Centre
- ☐ 其他 Others

如其他，请注明：
Others, detailed description:

声明：我将自觉遵守实验动物福利伦理原则，同意接受委员会或实验室管理者的监督与检查，如违反承诺和规定，自愿接受处罚。 Declaration: I will abide by the law and regulation stipulation, and accept the supervision and inspection by the committee and laboratory animal department. I will accept responsibility upon violation.

项目负责人签字（章）：[signature]
Signature (stamp) of PI

项目执行人签字（章）：
Signature (stamp) of Director of animal experiment

2024 年 8 月 28 日
Y M D

**伦理委员会审查意见：** 同 意<br>Approval opinion of Committee	
**主任委员签字（章）：**<br>Signature（stamp）of Chairman of Committee	**委员会签字（章）：**<br>Signature（stamp）of Committee<br>年 月 日<br>Y M D
